# Supplementary material for: Mesoglea biogenesis reveals a cryptic aboral valve for pressure regulation in cnidarian morphogenesis
Source: Sci Adv. 2026 Apr 17;12(16):eadz2530. doi: 10.1126/sciadv.adz2530 (PMC13089338; doi:10.1126/sciadv.adz2530)
Supplement: Supplementary file 1 — Figs. S1 to S5 Tables S1 to S3 Legends for movies S1 to S8 [file sciadv.adz2530_sm.pdf]

Science Advances
AAAS

Supplementary Materials for

Mesoglea biogenesis reveals a cryptic aboral valve for pressure regulation in cnidarian morphogenesis

Soham Basu *et al.*

Corresponding author: Aissam Ikmi, aissam.ikmi@embl.de

Sci. Adv. **12**, eadz2530 (2026)
DOI: 10.1126/sciadv.adz2530

The PDF file includes:

Figs. S1 to S5
Tables S1 to S3
Legends for movies S1 to S8

Other Supplementary Material for this manuscript includes the following:

Movies S1 to S8

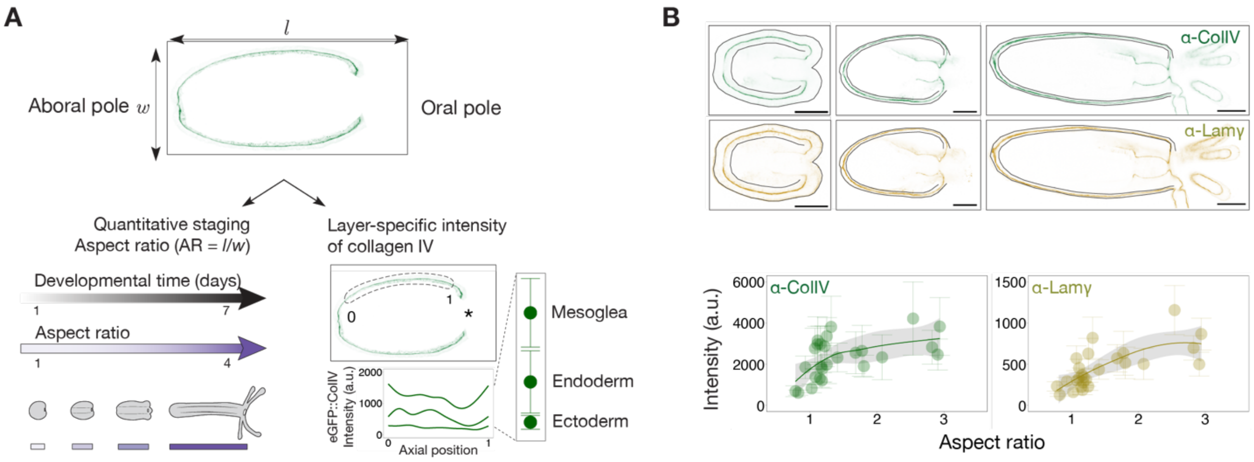

Figure S1: Quantification of Col IV and Laminin

(**A**) Schematic of developmental morphometrics and the regions used for quantifying eGFP::ColIV intensity.

(**B**) (Top) Confocal cross-sections of animals immunostained for Col IV and Laminin across developmental stages. Scale bar: 100µm. (Bottom) Quantification of extracellular Col IV and Laminin intensities as a function of body aspect ratio.

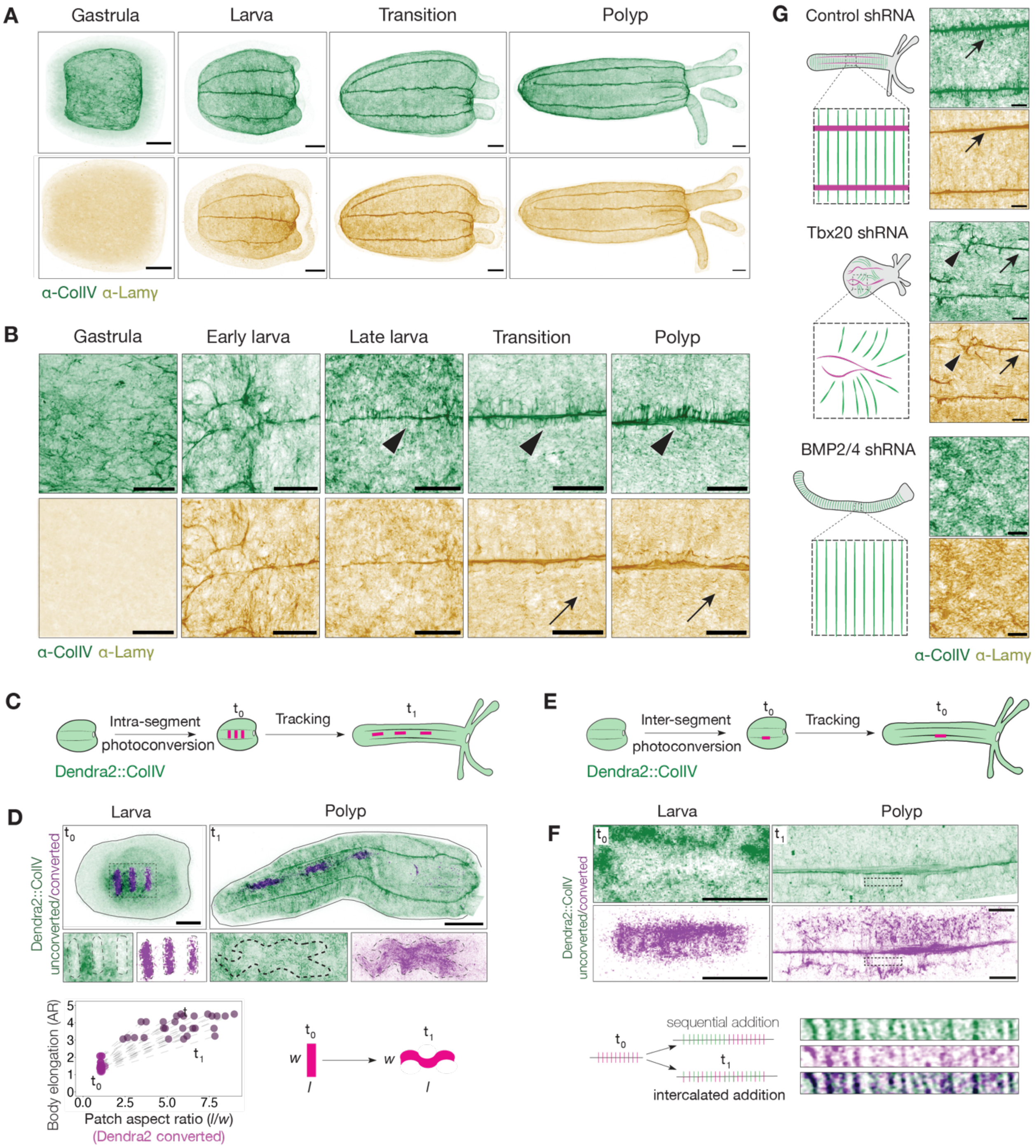

Figure S2: developmental dynamics of Col IV and Laminin

(**A**) Maximum intensity projections showing Col IV and Laminin immunostaining across developmental stages. Scale bar: 50µm.

(**B**) Magnified views of the regions shown in A, highlighting the spatial organization of Col IV and Laminin. Scale bar: 20µm.

(**C**) Schematic of the Dendra2::ColIV photoconversion assay in intra-segmental regions. Green: non-photoconverted; magenta: photoconverted.

(**D**) Time-course of photoconverted intra-segmental patches (magenta) from larva to primary polyp (*n* = 7 animals). Upper: Photoconverted regions at t_0 (day of conversion) and t_1 (3 days post-conversion). Lower: Zoom-in views of corresponding patches. Plot shows aspect ratio changes of tracked patches as a function of overall body elongation. Schematic summarizing the fate of intra-segmental Dendra2::ColIV photoconverted regions across development. Scale bar: 50 µm.
(**E**) Schematic of the Dendra2::ColIV photoconversion experiment in inter-segmental regions.
(**F**) Photoconverted inter-segmental patches (magenta) tracked from larva to primary polyp (*n* = 5 animals). Zoom-in shows the emergence of lateral Col IV bridges. Two models for lateral fiber addition are shown. Scale bar: 20 µm.
(**G**) Gene knockdown experiments disrupt endoderm morphogenesis and muscle organization (green: circular muscles; magenta: longitudinal muscles), showing resulting patterns of Col IV (green) and Laminin (yellow). *n* = 6 animals for control shRNA; *n* = 6 animals for Tbx20 shRNA; *n* = 6 animals for BMP2/4 shRNA. Scale bar: 20 µm.

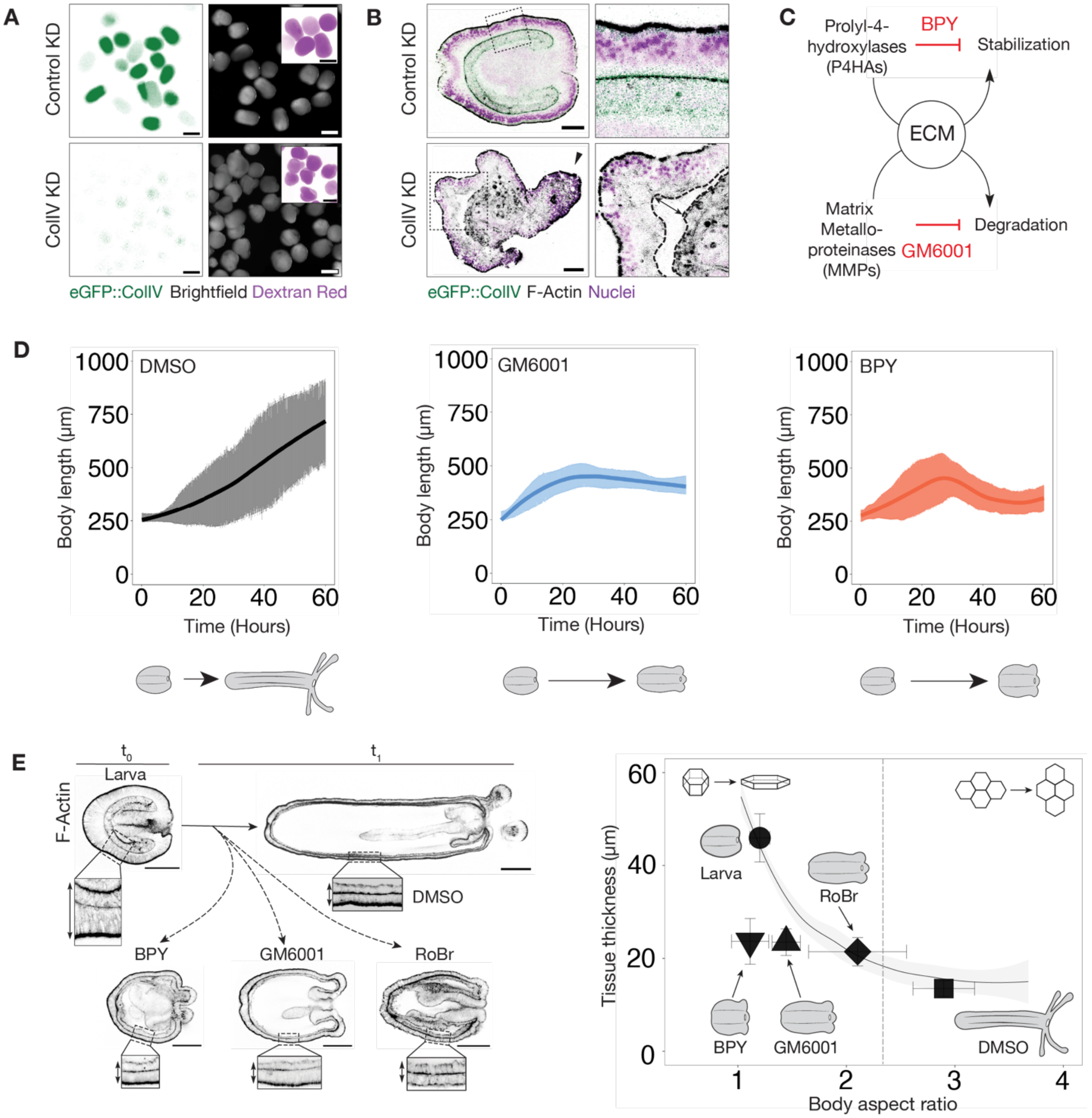

Figure S3: ECM perturbation and elongation dynamics

(A) Epifluorescence and brightfield images of larvae at 3 DPF from an eGFP::ColIV heterozygous cross with wild type, following control or *ColIV* shRNA injection. In control knockdown (KD) animals show normal morphology (n = 20), with ~50% of larvae expressing eGFP::ColIV as expected. In contrast, *ColIV* KD animals display a marked reduction in eGFP::ColIV signal and abnormal morphology (n = 47/51). Scale bar: 200 µm.

(B) Confocal cross-sections of representative control and Col IV KD larvae stained for F-actin and Hoechst (n = 8 each). Note the disorganized tissue architecture (arrowhead) and pronounced gap between ectoderm and endoderm (double-headed arrow) in the Col IV KD condition. Scale bar: 50 µm.

(C) Schematic summarizing pharmacological inhibitors targeting ECM stabilization and degradation.
(**D**) Plots showing changes in body length over time for each treatment condition. Solid lines represent the mean, and shaded areas indicate the standard deviation.
(E) (left) Mid-plane optical sections of animals stained for F-actin. Zoom-in views highlight alterations in body wall architecture under each treatment condition. Scale bar: 100μm. (Right) Quantification of body wall thickness as a function of body aspect ratio across all conditions. The black curve and light gray shading represent the mean and standard deviation of thickness measurements during normal development. *n* = 20 DMSO, *n* = 20 BPY, *n* = 20 GM6001, and the muscle anesthetic Rocuronium bromide (*n* = 20 RoBr).

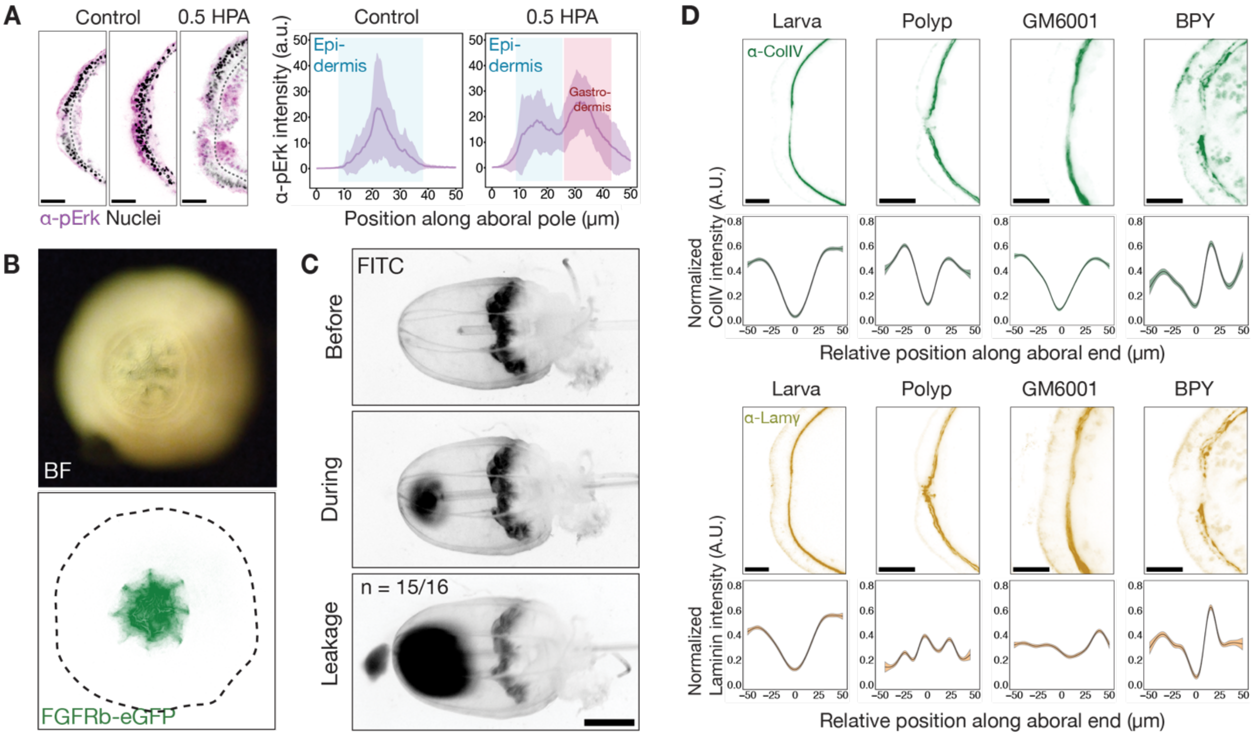

Figure S4. Profiles of pErk, Col IV, and Laminin at the aboral pole, and the presence of the aboral valve in adult polyps.

(A) (Left) Cross-sectional images of control polyps stained for pErk and Hoechst, showing two representative patterns of pErk signal intensity. Additional panel shows pErk staining 0.5 hours post-ablation (HPA) of the aboral pole. Scale bar: 20 µm. (Right) Quantification of pErk intensity in control versus ablated animals. Purple curve and light shading represent the mean and standard deviation. Signal localization within the epidermis versus endodermis is indicated.

(**B**) (Top) Brightfield (BF) image of the aboral pole in an adult. (Bottom) Aboral view of the FGFRb-eGFP expressing cells marking the aboral valve.

(**C**) Cavity inflation assay in an adult showing three representative time points: before inflation, during inflation, and at the point of leakage. The number of polyps exhibiting aboral leakage is indicated. Scale bar: 1mm.

(**D**) Cross-sectional images showing Col IV and Laminin immunostaining at the aboral end across the indicated treatment conditions. Plots show the spatial distribution of Col IV and Laminin intensities at each condition; solid lines represent the mean. Scale bar: 10 µm.

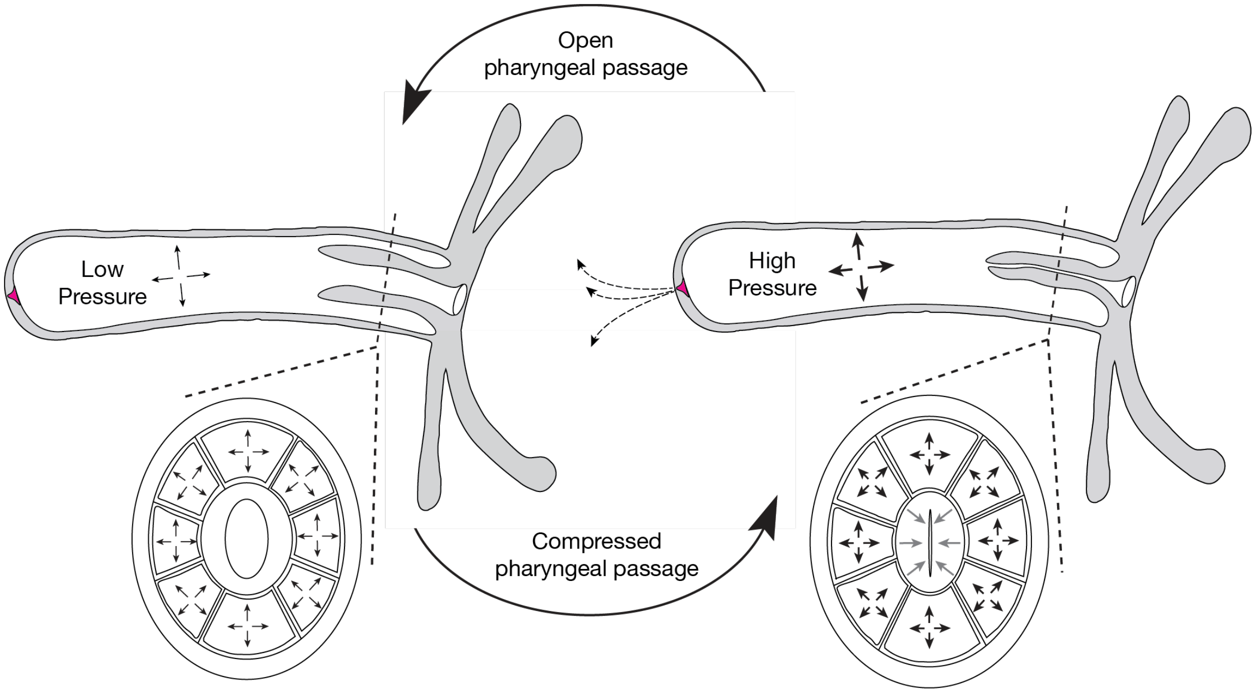

Figure S5. Working model of pharyngeal states under low and high internal cavity pressure. The schematic illustrates a primary polyp and a cross-section of the pharyngeal region. Note that the pharynx is connected to the body wall via eight mesenteries, forming eight fluid-filled pouches that are directly continuous with the main body cavity. Under low internal pressure, the pharyngeal passage remains open, permitting potential flow through the oral opening. In contrast, elevated pressure within these pouches compresses the pharynx, effectively sealing the passage and preventing fluid release through the oral end. Under this high-pressure scenario, the aboral opening functions as a release valve, enabling pressure relief through the aboral end.

Table S1. Reported secondary openings in cnidarians

Genus / Clade	Reference	Pore name & location	Proposed function	Mechanism of opening	Developmental onset of opening
Hydra (Hydrozoa)	Shimizu et al. 2007 (*33*)	Aboral pore at center of pedal/basal disc	Outward egestion of digested material	Not reported	late stage of Budding
Aequorea (Hydrozoa)	Aria & Chan, 1989 (*34*)	(i) Radial canal pores in gonadal region; (ii) Marginal papillae at tentacle bulbs	Radial pores eject large particles; papillae expel fine particulate residues	Not reported	Not reported
Halammohydra (Hydrozoa)	Polte and Schmidt-Rhaesa, 2011 (*35*)	Aboral opening of the adhesive organ	Not reported	Not reported	Not reported
Cotylorhiza (Scyphozoa)	Motta et al. 2025 (*36*)	(i) Proximal oral-arm openings on the inner, flattened canal (ii) Distal oral-arm openings on the outer, dilated canal.	(i) Intake of seawater and suspended food particles (ii) Egestion of digestive residues	Not reported	Not reported
Leptoseris (Anthozoa)	Schlichter 1991 (*37*)	Oral epithelial micropores (1–2 µm) on sclerosepta crests	Outflow of water; flow-through filtration; retention of suspended picoplanktonic food	Not reported	Not reported
Cerianthus (Anthozoa)	Child 1903 (*6*), Hyman 1940 (*38*)	Aboral pore	Water expulsion during rapid contraction	Not reported	Not reported
Edwardsia (Anthozoa)	Hyman 1940 (*39*)	Aboral pore	Not reported	Not reported	Not reported

Haloclava (Anthozoa)	Hyman 1940 (*39*)	Aboral pore	Not reported	Not reported	Not reported
Arachnanthus (Anthozoa)	Stampar et al 2018 (*40*)	(i) Tentacular pores (ii) Aboral pore	Not reported	Not reported	Not reported
Nematostella (Anthozoa)	Amiel et al 2015 (*41*)	Aboral pore	Not reported	Not reported	Not reported
	This study	Aboral valve	Hydraulic pressure release	Pressure-sensitive muscular valve and transient epidermal ruptures	Late larva-polyp transition

Table S2: Primary antibodies used for immunostaining and their working dilutions

**Antibody**	**Source**	**Dilution**
Anti-phospho-Myosin Light Chain	Cell Signaling Technology #3671S	1:50
Anti-eGFP	MBL #598S	1:500
Anti-eGFP	Abcam #ab1218	1:500
Anti-Laminin-γ	Bergheim et al., 2025 (*19*)	1:400
Anti-ColIV - JK2	Gift from Haruko Tomono	1:400
Anti-phospho-p44/42 MAPK (Erk1/2)(Thr202/Tyr204)	Cell Signaling Technology #4370	1:250
Anti-α-tubulin,	Sigma #T9026	1:250

Table S3: **Primer sequences used for shRNA synthesis**

**shRNA**	**Direction**	**Sequence**
GFP (control)	Forward	TAATACGACTCACTATAGGGGCACAAGCTGGAGTACAATTCAAGAGATTGTACTCCAGCTTGTGCCCTT
	Reverse	AAGGGCACAAGCTGGAGTACAATCTCTTGAATTGTACTCCAGCTTGTGCCCCTATAGTGAGTCGTATTA

Tbx20	Forward	TAATACGACTCACTATAGGGAACAGCTGCTTAAACATTC AAGAGATGTTTAAGCAGCTGTTCCCTT
	Reverse	AAGGGAACAGCTGCTTAAACATCTCTTGAATGTTTAAGC AGCTGTTCCCTATAGTGAGTCGTATTA
BMP2/4	Forward	TAATACGACTCACTATAGGACTGGATATTCAAGTGATTC AAGAGATCACTTGAATATCCAGTCCTT
	Reverse	AAGGACTGGATATTCAAGTGATCTCTTGAATCACTTGAAT ATCCAGTCCTATAGTGAGTCGTATTA
Col IV	Forward	TAATACGACTCACTATAGGGTGCAATGGTACTACAATTC AAGAGATTGTAGTACCATTGCACCCTT
	Reverse	AAGGGTGCAATGGTACTACAATCTCTTGAATTGTAGTAC CATTGCACCCTATAGTGAGTCGTATTA

Captions for Movies

Movie S1: Live imaging of early eGFP::ColIV embryos using confocal and light-sheet microscopy. Part 1: confocal time-lapse of multiple embryos undergoing gastrulation. Part 2: light sheet time-lapse of a single gastrulating embryo.

Movie S2: Live imaging of larva-polyp transition under the indicated conditions. Part 1: DMSO-control, part 2: GM6001-treated, and part 3: BPY-treated animals.

Movie S3: Live imaging of aboral leakage. Part 1: DMSO-control and part 2: BPY-treated animals.

Movie S4. Segmented FIB-SEM data of the aboral pole**.** Part 1: XY view, with oral side at the top and aboral side at the bottom. Note the unique structure of the mesoglea visible at 00:08. Part 2: XZ view, progressing from the epidermis to the gastrodermis. Observe the basal epidermal gap beginning at 00:17.

Movie S5: 3D rendering showing the dual conformational states of the muscular valve labeled with *FGFRb-eGFP*. Part 1: closed state, Part 2: open state and Part 3: Localisation of pMLC at the muscular valve.

Movie S6: Live imaging of cavity inflation in developing and adult polyps. Part 1: larva, part 2: early transition, part 3: late transition, part 4: primary polyp, and part 5: adult.

Movie S7: Egestion in primary polyps and adults. Part 1 shows egestion through the oral opening in primary polyps (n = 22). Part 2 shows oral egestion in adults (n = 6).

Movie S8: Live imaging of cavity inflation in the *FGFRb* mutant and its sibling. Part 1: a sibling of the *FGFRb* mutant (control), part 2: early response of the *FGFRb* mutant, and part 3: late response of the *FGFRb* mutant.
